# The microbiomes and metagenomes of forest biochars

**DOI:** 10.1038/srep26425

**Published:** 2016-05-23

**Authors:** Genevieve L. Noyce, Carolyn Winsborough, Roberta Fulthorpe, Nathan Basiliko

**Affiliations:** 1 Department of Geography, University of Toronto, Toronto, ON, Canada; 2 Department of Physical and Environmental Sciences, University of Toronto Scarborough, Toronto, ON, Canada; 3 Department of Geography, University of Toronto Mississauga, Mississauga, ON, Canada; 4 Department of Biology and the Vale Living with Lakes Centre, Laurentian University, Sudbury, ON, Canada

## Abstract

Biochar particles have been hypothesized to provide unique microhabitats for a portion of the soil microbial community, but few studies have systematically compared biochar communities to bulk soil communities. Here, we used a combination of sequencing techniques to assess the taxonomic and functional characteristics of microbial communities in four-year-old biochar particles and in adjacent soils across three forest environments. Though effects varied between sites, the microbial community living in and around the biochar particles had significantly lower prokaryotic diversity and higher eukaryotic diversity than the surrounding soil. In particular, the biochar bacterial community had proportionally lower abundance of *Acidobacteria, Planctomycetes*, and *β-Proteobacteria* taxa, compared to the soil, while the eukaryotic biochar community had an 11% higher contribution of protists belonging to the *Aveolata* superphylum. Additionally, we were unable to detect a consistent biochar effect on the genetic functional potential of these microbial communities for the subset of the genetic data for which we were able to assign functions through MG-RAST. Overall, these results show that while biochar particles did select for a unique subset of the biota found in adjacent soils, effects on the microbial genetic functional potential appeared to be specific to contrasting forest soil environments.

The use of biochars (pyrolyzed organic matter) as a soil amendment is rapidly increasing in popularity in managed ecosystems across the world as a means of improving soil fertility, particularly by raising soil pH and increasing nutrient availability, while also enhancing carbon (C) sequestration due to the recalcitrant structure of the biochar particles. Most field trials have been conducted in agricultural systems, including grain fields, rice paddies, and vineyards (e.g.^1^,^2^,^3^,^4^) and only a few trials have been implemented in forests (e.g.^5^,^6^,^7^). Consequently, the long-term effects of biochars on the function of the soil microbial community, especially in temperate forested ecosystems, are still unknown.

Biochar particles have often been hypothesized to provide a unique habitat for a subset of the soil microbial community due to their porous structure and adsorption of organic matter and nutrients^8^,^9^,^10^,^11^. However, biochars have extremely variable properties, depending on their feedstock and pyrolysis temperature. Although biochars are expected to be a particularly good habitat for bacteria, actinomycetes, and arbuscular mycorrhizal fungi^9^,^10^, this depends on the distribution of pore sizes within the particles. Nonetheless, several recent studies have used microscopy to document the fungal and bacterial colonization of the surface and interior pores of a wide range of biochar particles pyrolyzed from grass, straw, corn stover, and a variety of wood chips at temperatures ranging from 250 to 700 °C in both laboratory and field experiments^11^,^12^,^13^,^14^,^15^, providing evidence that soil microorganisms can use the interior of biochar particles as a habitat. However, most biochar studies have only looked at the initial colonization of particles, whereas it is becoming apparent that effects of biochars in soil systems are frequently transient; strong phylogenetic and functional microbial responses in the initial weeks or months after addition are often negligible after 12 months or longer^1^,^16^,^17^,^18^,^19^,^20^.

Specific knowledge of the microbes that colonize biochar particles is particularly limited as, to the best of our knowledge, only one study has implemented high-throughput marker gene sequencing community structure approaches on the biochar particles themselves, rather than less-precise techniques, and that study focused only on bacterial communities in Amazonian dark earth^21^. Instead, most previous work has only used high-throughput sequencing to compare soil with biochars to soil without biochars (e.g.^3^,^22^,^23^,^24^,^25^). Consequently, much remains to be learned about the biotic communities of biochars and their metabolic potential in other soil systems. Our work represents the first study to apply high-throughput sequencing to aged biochar particles collected from northern forests, as well as the first to apply environmental metagenomics to aged biochar particles from any ecosystem.

This study tested the hypothesis that biochar particles provide a unique microbial habitat, through examination of the structure and genetic functional potential of the forest soil microbial community living in aged biochar particles. We implemented a unique experimental design to directly contrast biochar particles with the surrounding soil by applying a combined approach of high-throughput amplicon sequencing (16S and 18S rRNA genes) and shotgun metagenomics to four-year-old biochar particles and adjacent soils in three environments of the Great Lakes‒St. Lawrence and Carolinian forest ecoregions of Central and Southern Ontario, Canada. We used this dataset to address three main questions: (1) Does forest biochar support a microbial community that is different from the surrounding soil community? (2) Does the functional potential of these microbial communities differ? (3) Is variability between the microbial communities of forest soil and biochar particles consistent across forests with contrasting soil chemistry?

## Results

### Sequence statistics

Total sequence depth for the 18 amplicon samples was 588,552 sequences for 16S amplicons and 285,815 sequences for 18S amplicons. The average numbers of quality sequences per treatment are given in Supplementary Table S1. The six metagenomic samples each had 1160 to 1960 Mbp of sequences (see Supplementary Table S2). Of the quality reads, 19.6 to 24.3% could be functionally annotated by the MG-RAST pipeline (Supplementary Table S2), which is similar to other forest soil metagenome studies^26^,^27^,^28^. On average, 19.7% of the soil reads were annotated compared to 23.3% of the biochar reads (Supplementary Table S2). Bacterial sequences predominated, with an average of 97.7% of total soil sequences and 97.2% of total biochar sequences matched to bacterial genomes in MG-RAST.

### Phylogenetic community composition

**Figure 1:**
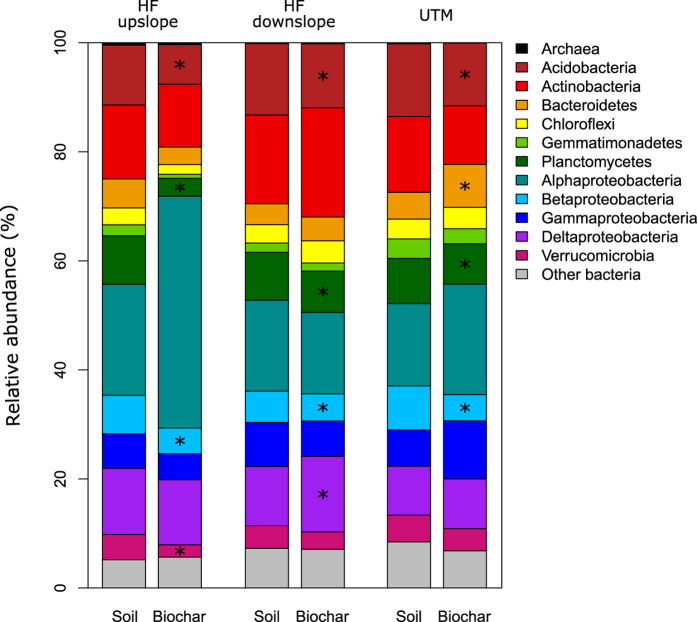
Relative contributions of archaea and bacterial phyla to total prokaryotic community DNA extracted from soil and biochar samples from three forest sites. *indicate phyla with significant (*p* < 0.05) differences in abundance between biochar particles and soil samples for the three replicates at each site.

Prokaryotic diversity was significantly lower in all biochar samples compared to the soil samples from the same forest, though these differences were stronger for the Chao1 index than for Faith’s PD (Table 1). Prokaryotic communities in all samples were dominated by *Acidobacteria, Actinobacteria, α-Proteobacteria* and *δ-Proteobacteria*, comprising 51 to 73% of all 16S sequences (Fig. 1). In all three forests, taxa belonging to *Acidobacteria, Planctomycetes*, and *β-Proteobacteria* were significantly lower (*p* < 0.05) in biochar samples compared to soil samples, whereas trends in other phyla varied by forest environment (Fig. 1). Relative abundances of *Acidobacteria, Planctomycetes*, and *β-Proteobacteria* orders are provided in Supplementary Table S3. At the dry, upland Haliburton Forest site (Great Lakes‒St. Lawrence forest), biochar particles had a lower abundance of *Verrucomicrobia* compared to the soil, whereas at the wet, downslope Haliburton Forest site biochar had higher abundance of *δ-Proteobacteria* (Fig. 1). At the UTM site (Carolinian Forest), biochar samples had a higher abundance of both *Bacteroidetes* and *Archaea* taxa (Fig. 1). Soil prokaryotic diversity was negatively correlated with total soil C (Chao1: *r* = −0.99, *p* = 0.024; Faith’s PD: *r* = −0.99, *p* = 0.026).

Of the 5190 prokaryotic taxa across all chars and soils, 118 taxa were unique to soils, i.e. not found in char; these taxa were mainly members of the *α-Proteobacteria, Planctomycetes, Actinobacteria, Acidobacteria*, and *δ-Proteobacteria*. In contrast, only six taxa were found solely in the biochar particles: two *Proteobacteria (Myxococcacea* and *Coxiellaceae* families), an *Actinobacteria (Solirubrobacterales* order), and unknown taxa from the *Chlorobi, Chloroflexi*, and *TM6* phyla. Though the majority of these taxa only composed 0.01 to 0.04% of the biochar prokaryotic communities, on average, the *Coxiellaceae* taxa composed 0.9% of the upslope Haliburton Forest biochar community and the *TM6* taxa composed 1.5% of the downslope and 2.2% of the upslope biochar communities. Five of these taxa were closely related to unknown bacteria found in other soil environments, implying that they are not always excluded from soils (see Supplementary Fig. S1).

**Figure 2:**
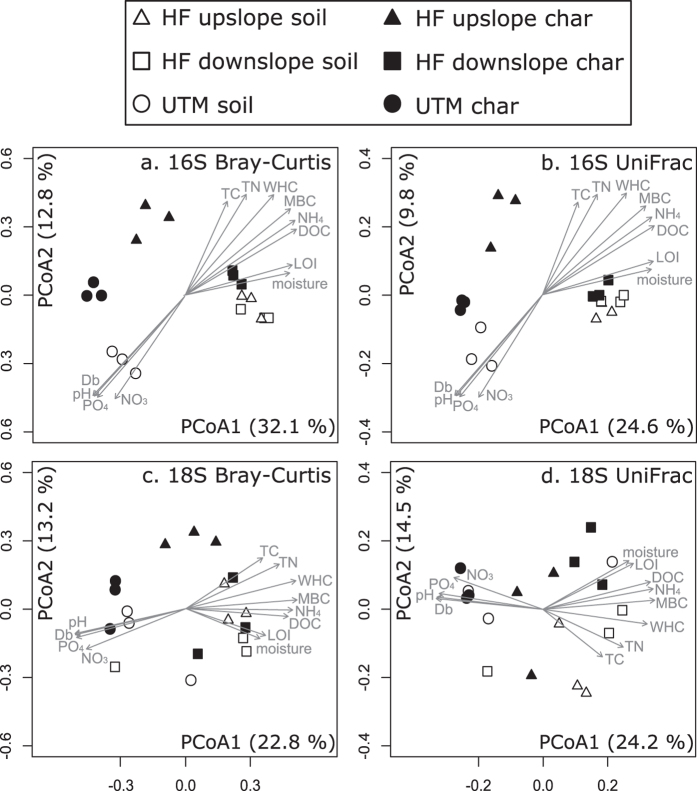
Principal coordinates analysis of (**a**) Bray-Curtis and (**b**) UniFrac distances of 16S amplicon data and (**c**) Bray-Curtis and (**d**) UniFrac distances of 18S amplicon data. White points are soil samples, grey black points are biochar samples, and shapes indicate forest site. Treatment effects (assessed through PERMANOVA) are shown in Table 2. Vectors represent soil chemistry variables that were significantly (*p* < 0.05) correlated with the PCoA results.

Sample type (biochar vs. soil) and forest site were significant factors for both Bray-Curtis and UniFrac distance matrices (Table 2; Fig. 2). For UniFrac ordinations, which take into account phylogenetic relationships between taxa, the divisions between soil and biochar communities were less distinct at UTM and the wet, downslope Haliburton Forest site, but biochar and soil communities from the upslope Haliburton site were still clearly separate (Fig. 2). Forest effects were driven by large variations in soil chemistry between the sites (Fig. 2; Table 3).

**Figure 3:**
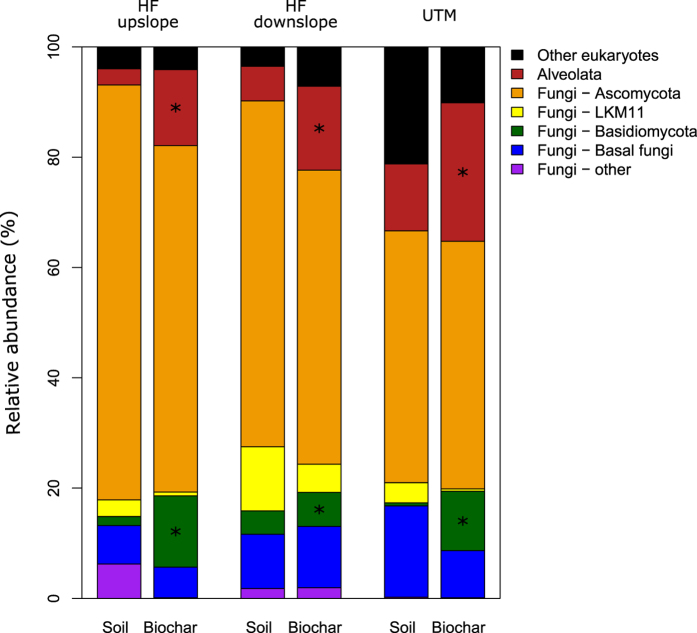
Relative contributions of eukaryotic groups to total eukaryotic community DNA extracted from soil and biochar samples from three forest sites. *indicate groups with significant (*p* < 0.05) differences in abundance between biochar particles and soil samples for the three replicates at each site.

Eukaryotic diversity was always higher in the biochar samples compared to their paired soil samples (Table 1). On average, fungi composed 84% of the soil eukaryotic sequences and only 75% of the biochar taxa (Fig. 3). Within the fungi, *Basidiomycota* had higher relative abundance in biochar particles compared to soil samples (*p* = 0.04; Fig. 3). Biochar particles from all sites contained many sequences in the *Aveolata* superphylum (mainly *Apicomplexa* and *Ciliophora*); these protists made up 18% of the biochar eukaryotic community but only 7% of the soil community (*p* < 0.01; Fig. 3). Unlike with prokaryotes, there were no site-specific differences in eukaryotic composition between biochar and soil samples. While only one taxon was unique to the soils (a *Mucoromycotina*), it composed 2.2% of the upslope Haliburton Forest eukaryotic community. Four taxa were found exclusively in the biochar particles: three protists (*Amastigomonas* sp, *Ripidomyxa* sp, and a member of the *Eimediidae* family) and one fungus (*Mucor* sp), but they all composed only 0.01 to 0.02% of the total community. Soil eukaryotic diversity (Faith’s PD) was negatively correlated with total soil N (*r* = −0.99, *p* = 0.048).

As with prokaryotes, forest site was a significant factor for both types of distance matrices (Table 2, Fig. 1). In contrast, sample type was only significant for Bray-Curtis (Table 2, Fig. 1). This was particularly true at the Haliburton Forest sites (Fig. 1). Differences in soil eukaryotic community composition between sites were again strongly correlated with measured aspects of soil chemistry (Fig. 2; Table 3).

### Functional potential of communities

The subsystem groups of carbohydrates, clustering-based subsystems (CBSS), and amino acids and derivatives accounted for the majority of functional genes in all metagenomes (see Supplementary Fig. S2). CBSS includes genes that are functionally coupled, but often have an unknown function. The six metagenomes showed minimal consistent differences in functional potential between biochar and soil samples (*p* = 0.08), but did vary across forest sites (*p* = 0.02). At the broadest subsystem level, the largest difference between biochar and soil samples was a higher abundance of carbohydrate metabolism functions in the soils (15.8% vs. 14.8%), but this difference was not significant (Supplementary Fig. S2).

**Figure 4:**
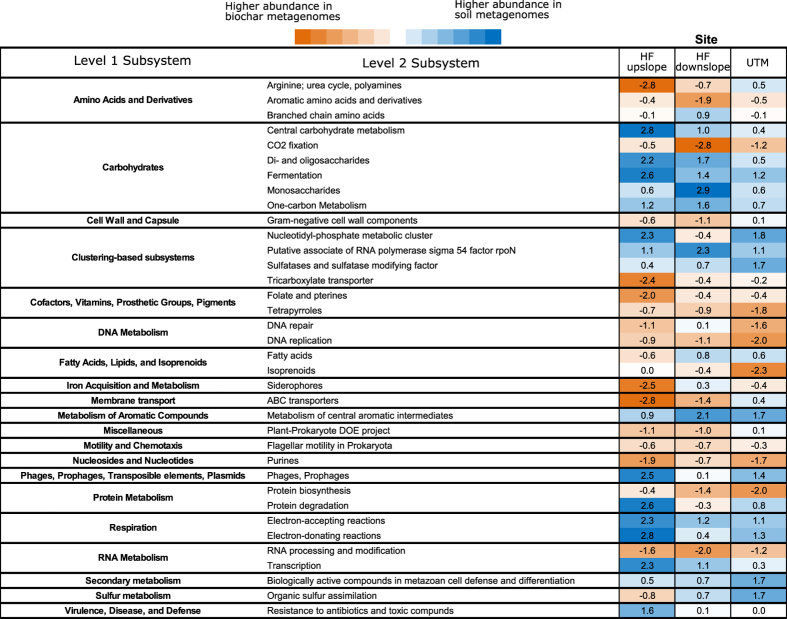
Level 2 Subsystems with the largest differences in relative abundance between soil and biochar samples at a single site. Values are differences in *z*-scores (relative abundance compared to the mean) between soil and biochar samples for each site. Negative values (orange boxes) indicate higher abundance in biochar metagenomes and positive values (blue boxes) indicate higher abundance in soil metagenomes.

Most differences in functional potential between soil and biochar samples were not consistent across all three sites (Fig. 4). The largest consistent effects included a higher abundance of genes associated with CO_2_ fixation and RNA processing and modification in the biochar metagenomes and of genes associated with fermentation, metabolism of central aromatic intermediates, and electron-accepting and electron-donating reactions in the soil metagenomes (Fig. 4). Similarly, there were almost no overall biochar versus soil effects on genes related to nitrogen (N), phosphorus (P), or sulphur (S) cycling (see Supplementary Fig. S3). The one exception was dissimilatory nitrite reductase genes, which were significantly less abundant in soil metagenomes compared to biochar metagenomes (*p* < 0.01) (Supplementary Fig. S3). Of the three sites, the upslope Haliburton Forest site had the largest overall differences in genetic functional potential between the biochar and soil microbial communities (Fig. 4).

### Comparison of taxonomic and functional diversity

Mantel tests indicated that there was a significant, but weak, positive correlation between prokaryotic and functional diversity across all biochar and soil samples (ρ = 0.32, *p* = 0.038), but there was no significant correlation between eukaryotic and functional diversity (ρ = 0.36, *p* = 0.083).

## Discussion

While previous studies have used microscopy to investigate how microorganisms colonize biochar particles^11^,^12^,^13^,^14^,^15^, and others have reported that biochars may select for communities conveying positive ecosystem attributes such as reduced greenhouse gas production (e.g.^29^,^30^), few have systematically compared biochar communities to bulk soil communities. Here, we showed that after four years in forest soil, biochar particles at these three contrasting forest sites clearly harboured distinct subsets of the soil prokaryotic and eukaryotic communities, implying that chemical or physical features of biochar particles select for certain taxa.

These results contrast with both prior studies in agricultural soils in which microbial colonization of wood-derived biochar particles was minimal after 56 days^14^ and three years^13^ and with a study by Taketani *et al*.^21^, in which the authors found that char particles removed from a forested Amazonian dark earth soil did not have distinct bacterial communities compared to the surrounding soil. This is likely due to the non-standard nature of biochars, i.e. characteristics that affect colonization may differ, as well as the variable nature of soil and the differences in exposure times. For example, agricultural soils typically have lower microbial diversity than heterogeneous forest soils^31^, so addition of biochar particles may have less selective effect than in forest soils. Similarly, pyrolysis temperature and initial feedstock will affect both the nutrient content and the structure of the biochar particles. Pyrolysis temperature is likely the most important control^30^, as it affects the pH, cation exchange capacity, organic C content, porosity, surface area, and structural heterogeneity of the biochar particles^32^. The biochar used by Quilliam *et al*.^13^ was pyrolysed at 450 °C, so the particles may have had a smaller surface area^30^, reducing the surfaces available for microbial colonization. When Mukherjee *et al*.^15^ investigated a high-temperature, wood-derived biochar, which is broadly similar to the 800 °C sugar maple biochar we used, they did observe abundant colonization after only 15 months in the field.

Previous work at Haliburton Forest has shown an initial release of potassium and phosphorus in the first few months after biochar was added to the soil^7^, so the biochar particles in this study likely initially provided an ideal habitat for bacterial taxa with copiotrophic growth strategies, due to pulses of labile C and other nutrients, and these taxa may have persisted over time. This theory is supported by the loss of phyla presumed to have oligotrophic growth strategies, such as *Acidobacteria, Verrucomicrobia*, and *Gemmatimonadetes* in the transition from the surrounding soil to the biochar particles, all of which generally decline in abundance in systems amended with organic C or N^33^,^34^,^35^. In addition, the largest biochar influences on bacterial communities occurred at the dry upland Haliburton Forest site, where the soil is acidic and P-deficient and thus the base cation influence and alkaline properties of the biochar particles likely provided a substantially different habitat than the surrounding soil. Similarly, the smallest effects occurred at the UTM site, which had the most alkaline soil.

However, some trends in the data do not fit with this explanation. For example, *β-Proteobacteria* were less abundant in the biochar of the upland Haliburton Forest site than in the soil, despite the hypothesis that these phyla are associated with environments with high nutrient availability^33^. Taketani *et al*.^21^ proposed that the recalcitrant nature of biochar means that the particles do not provide nutrients. Though that is in reference to Amazonian dark earth soils, in which the biochar is substantially older than in this study, it is likely that after four years in forest soils there are limited labile nutrients remaining in these biochar particles.

Other factors that might explain the phylum-level trends could include changes in water-holding capacity or pH, as these factors were significantly correlated with the distribution of soil microbial communities across the three sites. The higher water-holding capacity of the biochar compared to the UTM soil, and consequently the higher field moisture content, may have selected against microbial groups that typically thrive in dry environments, such as *Gemmatimonadetes*^36^. Similarly, the alkaline nature of the biochar particles may have selected against *Acidobacteria*, who generally thrive in acidic soils^37^. Taketani *et al*.^21^ also found that biochar particles had lower bacterial diversity than their surrounding soil and observed lower relative abundance of *Acidobacteria* in forest biochar particles compared to unamended soils, as in this study, though they did not find a similar decline in *Planctomycetes*^21^. However, the authors concluded that biochar vs. soil environments had a stronger effect on selecting for bacterial community composition than dominant vegetation type^21^, whereas we found that while biochar particles did have a strong selection effect on the bacterial community, it was secondary to the site-specific effect.

Though most high-throughput sequencing studies have focused on the effect of biochars on bacterial communities, our results indicate that biochar particles can substantially affect the eukaryotic community, especially by altering the presence of protists. Jin^11^ did investigate fungal diversity and also found lower eukaryotic diversity in the presence of biochar, but biochar effects on specific taxa varied between that study and ours. While we found that biochar particles had a higher relative abundance of *Basidiomycota* taxa, Jin^11^ found that biochar-amended soils contained fewer *Basidiomycota*. Though that study was conducted using 4-week incubations, the corn stover biochar was pyrolysed at 600 °C. In our case, the minimal colonization by fungi could indicate their inability to colonize the interior of the biochar particles^9^. However, Quilliam *et al*.^13^ found an equal presence of single-celled, filamentous, and hyphal microbes both in the middle of aged wood-derived particles and at the surface, indicating that microbes can colonize interior spaces of some biochars. Though the pyrolysis temperature for the biochar used here was 800 °C, compared to the 450 °C used by Quilliam *et al*.^13^, and pore size decreases with higher pyrolysis temperatures^9^, the average measured pore size for these particles of 50 and 90 μm indicates that these pores should be accessible by both bacteria^38^ and fungal hyphae^10^. However, the high abundance of protists associated with the biochar particles may have reduced bacterial abundance, due to increased grazing of bacteria.

Currently, two main theories have been proposed for the structuring of soil microbial communities. In niche theory, communities are structured by deterministic properties and taxa colonize niches based on their ecological traits^39^, as discussed above, following the “everything is everywhere, but, the environment selects” theory of Baas Becking^40^. In contrast, Hubbell’s neutral theory proposes that community structure is based only on dispersal mechanisms and stochastic properties^41^ and has been found to structure a variety of soil microbial communities^39^,^42^. As dispersal is particularly important for structuring soil microbial communities on small spatial scales^43^,^44^,^45^,^46^, the lower bacterial diversity in the biochar particles may by driven by these mechanisms. Mukherjee *et al*.^15^ have put forth a compelling case that microbial dispersal may play an important role in structuring bacterial communities after biochar addition, at least for specialist taxa. This theory could also explain the higher prevalence of protists associated with the biochar compared to the soil, since protists are very mobile in the soil matrix^43^.

It is important to caution that the choice of analytical methods can result in vastly different interpretations of community diversity or response to environmental gradients^44^,^47^. Here, we used two different metrics of between-site diversity and found that when phylogenetic-relatedness is accounted for, e.g. by using UniFrac instead of Bray-Curtis, the differences in microbial community composition between biochar and soil decreased. Similarly, differences in alpha diversity were also larger when using Chao1 compared to Faith’s PD, which also incorporates phylogeny. These effects were especially apparent for eukaryotic between-site diversity; sample type was not a significant factor for UniFrac-based eukaryotic diversity, implying that while the communities are distinct they are populated with closely-related organisms. However, the principal coordinate axes do only explain around 40% of the observed variation, so neither analysis is completely characterizing the microbial community composition.

According to our limited metagenomic analysis, the lower level of bacterial phylogenetic diversity in the biochar particles had no consequences for its genetic functional potential. Though DNA-analysis of biochar-associated communities can be challenging, due to the biochar particles potentially sorbing DNA fragments, because we observed clear biochar effects in the taxonomic data, it is unlikely that metagenome treatment effects would be missed from extraction methodological concerns. Instead, this observed stability and resilience of functional potential is likely a product of the high microbial community diversity in soils^48^,^49^ as the lower phylogenetic diversity in biochar relative to the surrounding soil may not have been a large enough “ecosystem shift” to disrupt the functional potential of the system. While greater microbial diversity may increase functional stability^50^, the theory of functional redundancy proposes that distinct taxa can often have the same functional abilities^49^,^51^,^52^. As many functional traits are consequently unrelated to phylogeny, studies have indicated that phylotypic diversity is often weak predictor of functional diversity^53^,^54^,^55^, which fits with the weak correlation between taxonomic and potential functional diversity found in this study.

Even though carbohydrate metabolism genes were significantly different in relative abundance between the biochar and soil metagenomes, the magnitude of these effects was small (<1%). This could indicate slight systematic differences in C availability and uptake between the biochar and soil samples, but the difference is extremely minor in comparison to other environmental metagenome studies. However, Jin^11^ did also find that the few microbes that colonized biochar particles after 3 years in agricultural soil had lower C use efficiency than microbes in the surrounding soil, so this slight trend may be worth further investigation.

Excluding ubiquitous assimilatory reduction, microbes involved in N, P, and S biogeochemical cycling compose a small subset of the soil microbial community compared to aerobic heterotrophs^56^. Nonetheless, previous studies using the same annotation database and a similar sequencing depth have found significant differences in N, P, and S cycling subgroups between different soil environments^27^,^57^, so our analysis should have also revealed any significant biochar effects on these processes if they existed. Previous work comparing biochar-amended soils to pristine soils has shown that addition of pine, grass, and greenwaste biochars can alter the abundance of microbes responsible for N cycling in soil mesocosms^58^,^59^,^60^ and increase microbial mediation of S and P availability for plants^61^. Microbes have also been shown to solubilize significant amounts of P from rice biochar in aqueous solutions^62^. However, biochar pyrolyzed at 800 °C, as used in this study, will contain less N and P than the lower-temperature biochars used in these previous experiments^63^ and thus may be expected to have a more minor impact on nutrient cycling. In addition, these prior experiments were all conducted over short time scales, when the biochars were releasing their initial pulses of nutrients, and in simplified systems, as opposed to long-term field trials.

There are, however, several key limitations to this study that should be considered. We used only one standardized biochar, but pyrolysis conditions and feedstock type are known to influence the biogeochemical role of biochars in soils^30^,^63^,^64^. Consequently, a productive direction for future work would apply these techniques to explore microbial communities inhabiting contrasting types of biochar in the same soil environment. Similarly, by only sampling after four years, we are unable to thoroughly describe how the observed communities formed over time. Subsequent work focusing on a time-series of particle colonization would be a valuable addition to the biochar literature and would likely clarify our hypotheses around the importance of dispersal mechanisms in structuring these communities. In addition, enclosing the char particles within mesh litterbags may have introduced artefacts, such as changes in moisture or exclusion of macrofauna. At the UTM site, which has an active earthworm populations, biochar particles inside the 1-mm mesh were more protected than the surrounding soil. The litterbags may also have prevented inward migration of large pieces of organic matter and differences in continuous nutrient input between the litterbags and the surrounding soil could affect the relative abundance of members of the soil microbial community. Furthermore, comparing only a subset of soil to a larger soil environment could potentially introduce artificial sampling effects due to the heterogeneity of the soil environment^65^,^66^, though our sampling protocol was designed to minimize this risk.

There are also several caveats surrounding the use of shotgun metagenomics, as discussed by Prosser^67^. In particular, the presence of a functional gene only offers limited information into what processes have the potential to occur within the soil environment and cannot be used to extrapolate to broader ecosystem functioning. Subsequent work would benefit from assessment of functional gene expression, using tools such as metatranscriptomics and metaproteomics, to expand the results presented here. Soil microorganism genes are also underrepresented in annotation databases^68^ and the databases are likely misannotating or missing key genes^69^. As only 20 to 25% of the metagenomes were able to be annotated, we must assume that the complete functional capacities of these communities were not captured in this analysis.

In summary, these results show that while biochar particles did select for a unique subset of the biota found in adjacent soils, effects on the microbial genetic functional potential appeared to be specific to contrasting forest soil environments; metagenomes in biochar and soil across two distinct terrestrial ecoregions and between upland and lowland environments were very similar relative to differences between soil types. Though we did observe variability in prokaryotic and eukaryotic diversity and community composition between biochar and soil samples, biochar effects on microbial community functional potential, although detectable, were minor and site-specific.

## Materials and Methods

### Site descriptions and experimental design

In fall 2010, 1-mm mesh bags (10 × 10cm) containing 15 g of biochar were distributed under the litter layer across three sites within two forest environments: the Haliburton Forest and Wildlife Reserve and the University of Toronto Mississauga (UTM) campus forest. Haliburton Forest is a tolerant hardwood forest dominated by sugar maple (*Acer saccharum*) in the Great Lakes‒St. Lawrence Region of Central Ontario, Canada with nutrient-poor shallow Dystric Brunisols overlying the granitic Precambrian Shield. The entire region is actively managed using single-tree selection silviculture and was last harvested in 2000. The two Haliburton Forest sites were a saturated, poorly-drained, downslope area (i.e. a small swamp) and a well-drained, upslope environment. Many Ontario forests, including Haliburton Forest, have soil conditions that are not ideal for tree growth, due to low soil pH and nutrient content. Biochar has been shown to increase soil organic C, N, and P concentrations, overall nutrient retention, pH, base cation saturation, soil cation exchange capacity, and water-holding capacity^30^,^32^,^38^, which means it can be a valuable amendment in these forested ecosystems. The UTM forest is a remnant, unmanaged Carolinian forest on Brown Luvisols with calcareous parent material. Earthworms (*Lumbricus terrestris*) are very abundant at this site, so there is only a transient litter layer. UTM litterbags were deployed in a sugar maple dominated grove, also in a well-drained, upslope environment. Characteristics of all three soils are given in Table 3. The 1-mm mesh size was chosen to balance potential particle loss with soil fauna exclusion artefacts. Between 5 and 10 percent of the biochar in the litterbags was lost in the first year of deployment, after which the mass in the litterbags remained relatively stable for the next four years (see Supplementary Table S4).

### Biochar synthesis

The biochar used at all sites was produced by a stoker grate-type gasifier operating in pyrolysis mode. Sugar maple was used as the feedstock, as it is the dominant species in these forests. Dried woodchips (approximately 2.5 × 5 cm) were screw-fed at 140 kg h^−1^ onto a 500 °C chain grate, through which air was fed upwards. Woodchips spent approximately 10 minutes in the furnace, during which they reached a maximum temperature of 800 °C. Biochar properties are listed in Table 4.

### Soil and biochar physicochemical characterization

Soil pH was measured in a 1:10 slurry of field-moist soil and distilled water. Biochar pH was measured in slurries of 10 g biochar to 75 mL 0.5 M K_2_SO_4_. Organic matter content was assessed using a loss-on-ignition (LOI) technique for both soil and biochar in which dried, ground samples were heated in ceramic crucibles at 550 °C for 6 h. Biochar samples were further heated at 750 °C for an additional 6 h to assess ash content. Water-holding capacity (WHC) was determined by saturating soil samples and then draining for 2 h, following the protocol of Priha and Smolander^70^. Field moisture content was determined by drying soil and biochar at 105 °C for 24 h. Microbial biomass C in the soils (MB-C) was assessed by chloroform fumigation followed by extraction with 0.5 M K_2_SO_4_ and analysis on a TOC/TN analyser as described previously by Pugliese *et al*.^71^. Total C, N, and S were measured by combustion analysis using a VarioMax CN analyser (Elementar Analysensystem, Germany) for C and N and an Eltra Helios (Verder Scientific,Germany) for S. 10-g subsamples of soil and biochar were extracted with 50 mL (soil) or 75 mL (biochar) of 0.5 M K_2_SO_4_ and the extracts were analysed for extractable organic C on a TOC/TN analyser (IL550, Lachat Instruments, USA). For Haliburton Forest soils, these extracts were also analysed for inorganic N and P on a flow injection analyser (FIA 8500, Lachat Instruments, USA) using Quik-Chem methods 31-107-04-1 and 31-115-01-1-H. For UTM soils, KCl-extractable inorganic N and Bray-1 available P were measured as described by Pugliese *et al*.^71^. Biochar Ca^2+^, K^+^, and Mg^2+^ content was determined through a sulphuric acid digest with selenium dioxide as a catalyst followed by analysis on an Genesis ICP-OES (Spectro Analytical Instruments, Germany). Biochar chip size distribution was determined by sequential sieving. A dissecting microscope was used to measure the diameters of visible pores in the four-year-old biochar. A minimum of three analytic replicates were conducted for all analyses.

### Sample collection and DNA extraction

In fall 2014, three biochar litterbags were collected from each of the three sites. All litterbags were still at the litter-mineral soil interface. Key properties of these aged biochar particles from each of the three sites are listed in Table 4. Visible pores ranged from 50 to 210 μm in diameter, with most pore diameters falling between 50 and 90 μm. Surface soil grab samples covering approximately a 10 × 10 cm area adjacent to the litterbags and at the same depth were also collected from each site. By using soil samples of equivalent volume to the biochar litterbags, we minimized potential effects that could arise from comparing a small subsample of biochar to a larger sample of surrounding soil. All samples were frozen at −20 °C until analysis. Community DNA was extracted in duplicate from all 18 samples using MoBio PowerSoil DNA extraction kits (MoBio Laboratories, USA), following the manufacturer’s instructions, and duplicates were pooled prior to further analysis. Using sterilized forceps, approximately 2 g of biochar particles were removed from the interior of the litterbag, to reduce edge effects and minimization contamination from soil. Visible roots were removed and the particles were finely ground before extraction. DNA extracts using only 0.25 g of biochar were not visible on a 1% agarose gel, so all extractions were done on 0.5 g biochar instead. Soil extractions were done with 0.25 g subsamples from homogenized soil samples.

### PGM sequencing of 16S and 18S rRNA gene amplicons

rRNA gene amplicon sequencing was conducted on all samples using an Ion Torrent PGM at Molecular Research LP (Shallowater, TX, USA) following the manufacturer’s guidelines. The V4 region of the 16S rRNA gene was targeted using primers 515f and 806r^72^ and a portion of the 18S rRNA gene was targeted for all eukaryotes using primers 7f and 570r. Primer sequences are given in Supplementary Table S5. Amplification conditions were as follows: initial denaturation for 3 min at 94 °C, 28 cycles of 94 °C for 30 sec, 53 °C for 40 sec, and 72 °C for 1 min, and final extension at 72 °C for 5 min.

Sequence data were processed in QIIME^72^ following a modified version of the UPARSE standard pipeline^73^,^74^. In summary, barcodes and primers were removed from the sequences and then, using USEARCH 7, sequences were filtered with a maximum expected error of 1, dereplicated, clustered to OTUs using the UPARSE method, and filtered for additional chimeras using the Gold database. Using the uclust method, taxonomy was assigned to representative 16S sequences using the Greengenes database (13_08) at 97% similarity and to representative 18S sequences using Silva (111) at 90% similarity. Sequences were aligned using the PyNAST algorithm. Additional filtering was performed in QIIME to discard sequences identified as chloroplasts or mitochondria from the 16S dataset and sequences identified as plants or algae from the 18S dataset. Final sequences were deposited at the European Nucleotide Archive (ENA, http://www.ebi.ac.uk.ena/) under accession number PRJEB10273. All samples were rarefied prior to downstream analysis. Chao1 and Faith’s phylogenetic distance (PD) were used to assess diversity within each sample (α-diversity). Pairwise relationships between communities (β-diversity) were determined using Bray-Curtis and unweighted UniFrac^75^.

### Shotgun metagenomics

Biochar and soil replicates from each site were pooled prior to metagenomics analysis, for a total of three biochar and three soil samples. Shotgun metagenomics was conducted on an Illumina HiSeq2000 platform (2 × 100 bp) at Molecular Research LP. Using MG-RAST, paired reads were merged with a minimum overlap of 8 bp and a maximum difference of 10%. After removal of artificial duplicate reads, unassembled DNA sequences were annotated by SEED subsystem category with the MG-RAST pipeline version 3.5^76^ using BlastX with an *e*-value cutoff of 1 × 10^−5^ and a minimum read length of 50 bp. Sequences were deposited in MG-RAST under ID numbers 4623593.3 to 4623598.3.

### Comparative statistical analyses

All statistical analyses were performed in R, using the vegan and labdsv packages. Taxonomic and phylogenetic relationships between samples were visualized using principal coordinate analysis (PCoA) of the Bray-Curtis and UniFrac dissimilarity matrices with vectors added to represent gradients in measured environmental variables. Pearson’s correlation coefficients were calculated to determine relationships between diversity indices, PCoA scores, and soil chemical variables. Permutational multivariate analysis of variance was used to assess the effects of sample type and forest site on community composition. T-tests were used to determine significant differences in phylogenetic community composition between biochar and soil samples for each site, with Bonferroni corrections for multiple tests. Mantel tests with Spearman’s correlation were used to compare pairwise differences between samples based on taxonomic and metagenomics data.

## Additional Information

**How to cite this article**: Noyce, G. L. *et al*. The microbiomes and metagenomes of forest biochars. *Sci. Rep.* **6**, 26425; doi: 10.1038/srep26425 (2016).

## Supplementary Material

Supplementary Information

## Figures and Tables

**Table 1:** Mean ± SEM Chao1 and Faith’s PD estimates of prokaryotic and eukaryotic diversity for biochar and soil samples.

	Prokaryotes			
	Chao 1		Faith’s PD	
	Soil	Biochar	Soil	Biochar
HF upslope	**1122 ± 25**	**785 ± 23**	**68.8 ± 0.8**	**56.8 ± 1.3**
HF downslope	**1224 ± 31**	**1122 ± 35**	74.2 ± 0.9	70.3 ± 1.3
UTM	**1266 ± 48**	**1065 ± 19**	76.4 ± 1.8	71.6 ± 0.6
	**Eukaryotes**			
	**Chao 1**		**Faith’s PD**	
	**Soil**	**Biochar**	**Soil**	**Biochar**
HF upslope	**281 ± 10**	**305 ± 12**	14.7 ± 0.3	16.4 ± 0.6
HF downslope	**318 ± 14**	**356 ± 11**	**17.0 ± 0.4**	**20.3 ± 0.4**
UTM	**349 ± 12**	**397 ± 6**	**16.1 ± 0.6**	**19.3 ± 0.4**

Bolded values indicate significantly different (*p* < 0.05) levels of diversity between biochar and soil samples from the same site.

**Table 2:** PERMANOVA results for prokaryote (16S) and eukaryote (18S) rRNA amplicons based on Bray-Curtis and UniFrac distance matrices (shown in Fig. 3).

	Prokaryotes	
	Bray-Curtis	UniFrac
Sample Type	F_1,12_ = 11.3; ***p*** = **0.002**	F_1,12_ = 6.38; ***p*** = **0.004**
Site	F_2,12_ = 21.8; ***p*** < **0.001**	F_2,12_ = 15.3; ***p*** < **0.001**
Sample × Site	F_2,12_ = 4.49; ***p*** = **0.009**	F_2,12_ = 3.37; ***p*** = **0.023**
	**Eukaryotes**	
	**Bray-Curtis**	**UniFrac**
Sample Type	F_1,12_ = 3.10; ***p*** = **0.028**	F_1,12_ = 2.13; *p* = 0.075
Site	F_2,12_ = 5.67; ***p*** = **0.002**	F_2,12_ = 9.32; ***p*** < **0.001**
Sample × Site	F_2,12_ = 0.938; *p* = 0.470	F_2,12_ = 1.10; *p* = 0.335

**Table 3:** Characteristics of the three study sites.

	HF upslope	HF downslope	UTM
Soil class	Dystric Brunisol	Orthic Humic Gleysol	Brown Luvisol
Soil texture (A horizon)	sandy-loam	sandy-loam	sandy clay loam
LFH/O depth (cm)	5–10	15	<2
pH (H_2_O)	4.9 ± 0.2	5.2 ± 0.2	6.8 ± 0.1
Bulk density (g cm^−3^)	0.79 ± 0.02	0.81 ± 0.03	1.03 ± 0.04^a^
LOI (%)	31.5 ± 3.4	87.6 ± 1.8	12.3 ± 1.0
WHC (g water g^−1^ dry soil)	2.3 ± 0.5	2.1 ± 1.2	0.87 ± 0.1
Field moisture content (g water g^−1^ dry soil)	2.1 ± 0.8	8.8 ± 2.1	0.48 ± 0.1
MB-C (mg C g^−1^ dry soil)	1.2 ± 0.06	1.5 ± 0.11	0.08 ± 0.04^a^
Extractable DOC (mg C g^−1^ dry soil)	1.3 ± 0.07	2.3 ± 0.13	0.09 ± 0.03^a^
Total C (%)	45.1 ± 0.4	19.3 ± 3.4	6.8 ± 1.2
Total N (%)	2.8 ± 0.2	1.7 ± 0.2	0.5 ± 0.1
Extractable NO_3_^−^ + NO_2_^−^ (μg N g^−1^ soil)	21.4 ± 2.5^b^	26.1 ± 6.3^b^	33.6 ± 2.5^a^,^c^
Extractable NH_4_^+^ (μg N g^−1^ soil)	180.8 ± 12.4^b^	278.8 ± 20.4^b^	7.55 ± 0.8^a^,^c^
Extractable PO_4_^3−^ (μg P g^−1^ soil)	8.2 ± 2.3^b^	15.6 ± 3.8^b^	78.6 ± 23.6^a^,^d^

Values represent means ± SEM for the top 5 cm of the soil.

^a^Data are from Pugliese *et al*.^71^

^b^K_2_SO_4_ extraction.

^c^KCl extraction.

^d^Bray-1 extraction.

**Table 4:** Biochar characteristics.

	Initial	Final		
		HF upslope	HF downslope	UTM
pH (in 0.5 M K_2_SO_4_)	10.6 ± 0.9	n/a	n/a	n/a
Dry bulk density (g cm^−3^)	0.12 ± 0.01	n/a	n/a	n/a
LOI (%)	93.9 ± 2.3	96.4 ± 0.8	82.9 ± 3.8	70 ± 3.9
Ash (%)	7.99 ± 2.3	2.65 ± 0.12	5.87 ± 2.6	15.5 ± 3.3
WHC (g water g^−1^ dry biochar)	2.2 ± 0.3	n/a	n/a	n/a
Field moisture content (g water g^−1^ dry biochar)	n/a	2.33 ± 0.37	3.20 ± 1.11	2.70 ± 0.79
Total C (%)	77	n/a	n/a	n/a
Total N (%)	0.24	n/a	n/a	n/a
Total S (%)	0.21	n/a	n/a	n/a
Extractable NO_3_^−^ + NO_2_^−^ (μg N g^−1^ biochar)	2.55 ± 0.13^b^	n/a	n/a	n/a
Extractable NH_4_^+^ (μg N g^−1^ biochar)	0.33 ± 0.09^b^	n/a	n/a	n/a
Extractable PO_4_^3−^ (μg P g^−1^ biochar)	11.8 ± 2.0^b^	n/a	n/a	n/a
Ca^2+^ (mg g^−1^ biochar)	23.8^a^	n/a	n/a	n/a
K^+^ (mg g^−1^ biochar)	8.0^a^	n/a	n/a	n/a
Mg^2+^ (mg g^−1^ biochar)	0.5^a^	n/a	n/a	n/a
Size distribution (% of total)				
<2 mm	15.4 ± 3.0	n/a	n/a	n/a
2–3.5 mm	14.3 ± 3.2	n/a	n/a	n/a
3.5–4.75 mm	16.8 ± 2.8	n/a	n/a	n/a
4.75–6 mm	17.3 ± 5.1	n/a	n/a	n/a
6–9.5 mm	20.5 ± 4.9	n/a	n/a	n/a
>9.5 mm	15.6 ± 4.6	n/a	n/a	n/a

Initial data encompasses the pre-experiment biochar characteristics; final data summarizes key characteristics of the biochar particles recovered from the three sites. Values represent means ± SEM.

^a^No replicates conducted.

^b^K_2_SO_4_ extraction.
