# Supplementary material for: The microbiomes and metagenomes of forest biochars
Source: Sci Rep. 2016 May 23;6:26425. doi: 10.1038/srep26425 (PMC4876420; doi:10.1038/srep26425)
Supplement: Supplementary Information [file srep26425-s1.doc]

SUPPLEMENTARY INFORMATION

The microbiomes and metagenomes of forest biochars

Genevieve L. Noyce, Carolyn Winsborough, Roberta Fulthorpe, Nathan Basiliko

**
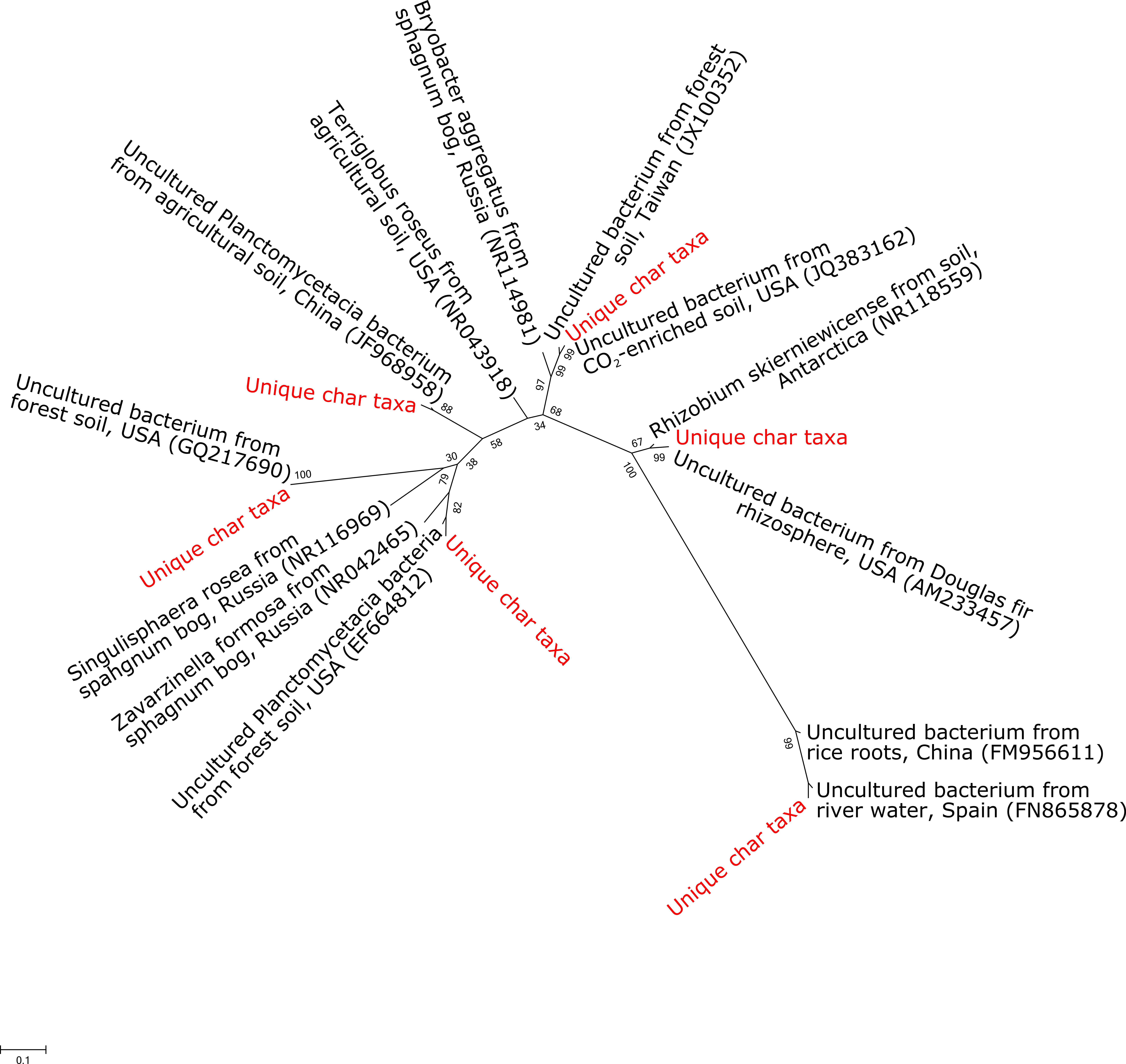
**

**Supplementary Figure S1**. Phylogenetic tree of unique bacterial OTUs from biochar and closely related organisms generated using the maximum likelihood method based on the Kimura 2-parameter model.


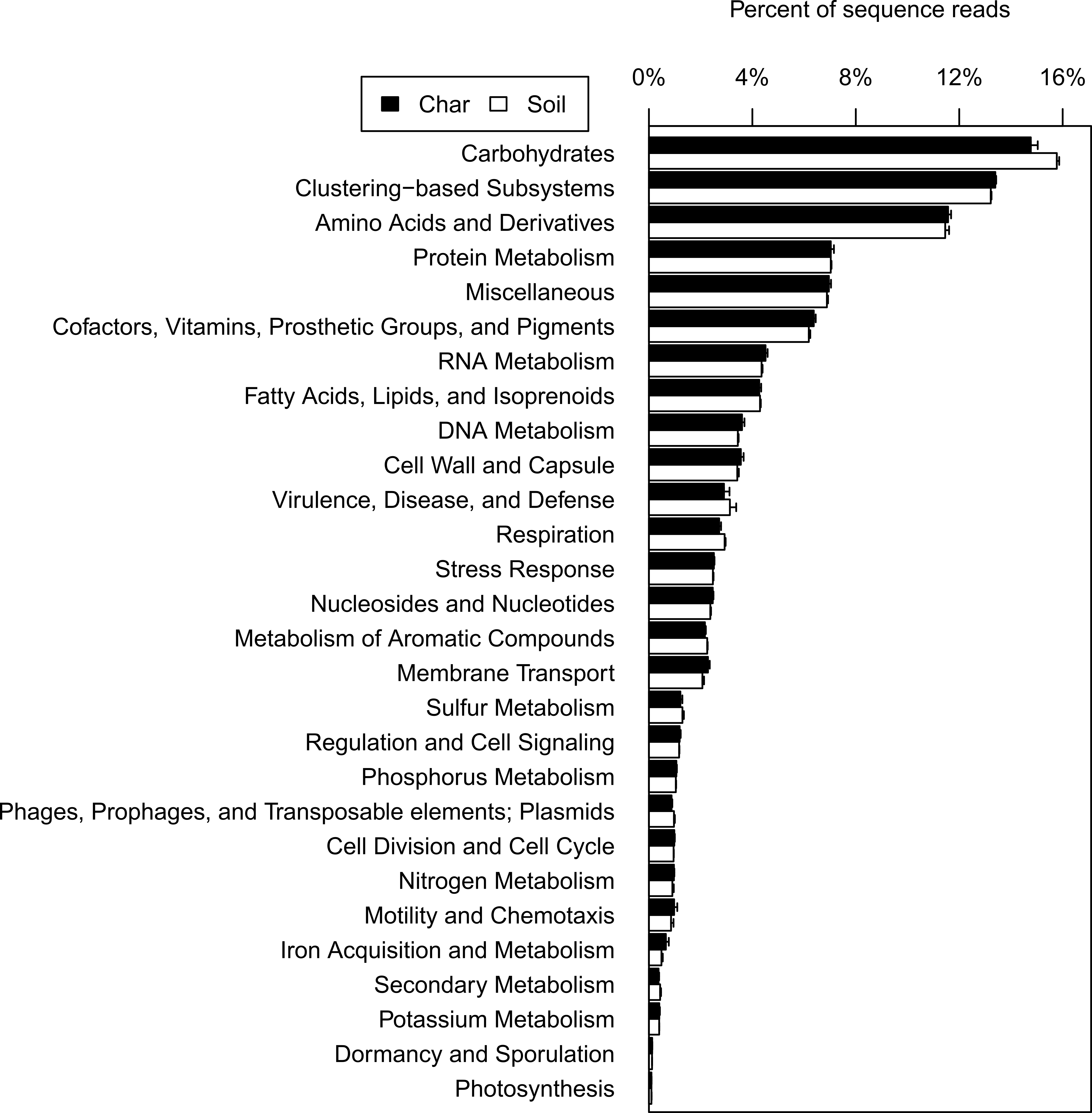


**Supplementary Figure S2**. Average abundance of genes in Level 1 functional groups for biochar (black) and soil (white) metagenomes. Error bars represent two standard error.

**
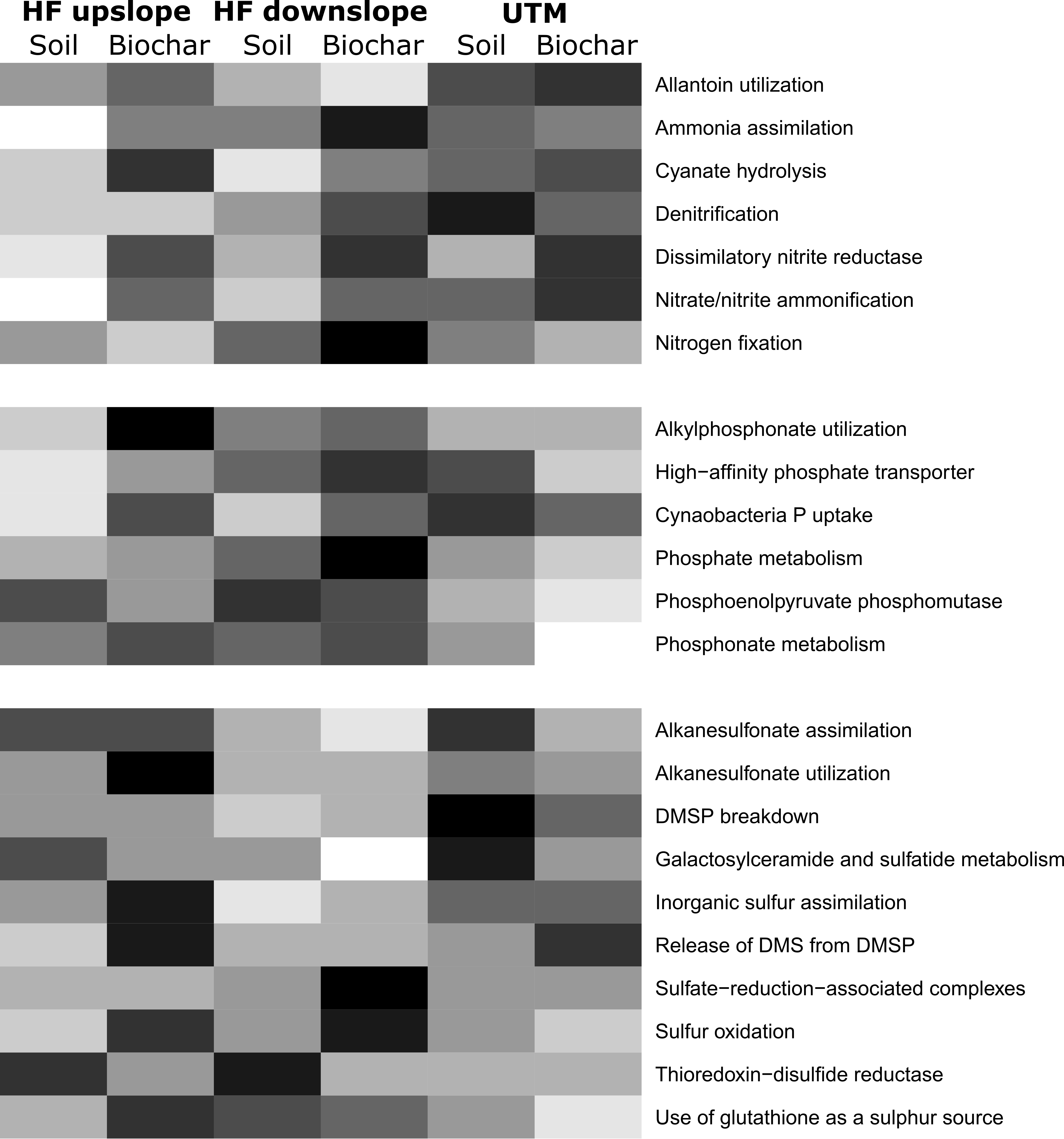
**

**Supplementary Figure S3**. Heatmaps indicating differences in relative abundance of functional genes involved in nitrogen (top), phosphorus (middle) and sulphur (bottom) cycling in the biochar and soil metagenomes. Rows are scaled to have a mean of zero and a standard deviation of one. Colors indicate the relative abundance of each gene across the six metagenomes ranging from highest (black) to lowest (white).

**Supplementary Table S1**. Percentage of sequencing reads remaining after quality control and average number of sequence reads per treatment for 16S and 18S datasets.

|  | **16S** | **18S** |
| --- | --- | --- |
| % seq. passing QC | 63.02 | 53.42 |
| No. quality seq. per treatment | | |
| HF upslope soil | 68,115 | 12,060 |
| HF upslope char | 19,482 | 13,956 |
| HF downslope soil | 49,586 | 15,730 |
| HF downslope char | 20,530 | 15,173 |
| UTM soil | 33,005 | 19,183 |
| UTM char | 5,464 | 19,167 |

**Supplementary Table S2**. Total number of shotgun sequencing reads, total number of Mbp, number of sequences passing quality control parameters, and percent of sequences functionally annotated.

| Sample | No. seq. reads | Total Mbp | No. seq. post-QC | % seq. annotated |
| --- | --- | --- | --- | --- |
| HF upslope soil | 15,301,392 | 1,553 | 13,900,454 | 19.7 |
| HF upslope char | 14,926,993 | 1,534 | 13,755,096 | 24.1 |
| HF downslope soil | 13,316,765 | 1,356 | 12,275,560 | 19.9 |
| HF downslope char | 13,067,297 | 1,331 | 11,704,046 | 24.3 |
| UTM soil | 19,293,337 | 1,959 | 17,601,295 | 19.6 |
| UTM char | 11.397,806 | 1,159 | 10,420,586 | 21.6 |

**Supplementary Table S3**. Relative abundance of orders composing *Acidobacteria*, *Planctomycetes*, and *β-Proteobacteria*.

|  | **HF upslope** | | **HF downslope** | | **UTM** | |
| --- | --- | --- | --- | --- | --- | --- |
|  | Soil | Biochar | Soil | Biochar | Soil | Biochar |
| **Acidobacteria** | | | | | | |
| *CCU21* | 1.19 | 1.28 | 1.24 | 0.78 | 1.48 | 2.23 |
| *iii1-15* | 20.04 | 13.54 | 11.94 | 13.44 | 17.47 | 32.55 |
| *Acidobacteriales* | 23.10 | 18.52 | 25.53 | 14.72 | 32.43 | 25.65 |
| *MVS-40* | 0.12 | 0.02 | 0.08 | 0.00 | 0.01 | 0.00 |
| *Ellin6513* | 25.10 | 32.16 | 24.23 | 38.24 | 19.49 | 13.91 |
| *Holophagales* | 0.26 | 0.00 | 0.01 | 0.00 | 0.00 | 0.00 |
| *JH-WHS99* | 0.08 | 1.08 | 0.11 | 0.13 | 0.10 | 0.11 |
| *Solibacterales* | 22.11 | 26.25 | 27.23 | 23.75 | 17.80 | 20.14 |
| *Sva0725* | 1.82 | 0.15 | 0.46 | 0.92 | 2.01 | 0.67 |
| *11-24* | 0.19 | 0.00 | 0.02 | 0.02 | 0.01 | 0.00 |
| *DS-100* | 0.03 | 0.00 | 0.00 | 0.00 | 0.00 | 0.00 |
| *Ellin7246* | 0.00 | 0.00 | 0.00 | 0.00 | 0.14 | 0.11 |
| *PK29* | 0.13 | 0.71 | 0.03 | 0.00 | 0.20 | 0.72 |
| *RB41* | 3.49 | 4.22 | 1.51 | 3.99 | 4.56 | 1.34 |
| *32-20* | 0.36 | 0.02 | 1.37 | 1.25 | 0.21 | 0.11 |
| *DS-18* | 0.59 | 0.07 | 5.03 | 0.54 | 2.24 | 0.50 |
| *SJA-36* | 0.00 | 0.00 | 0.03 | 0.04 | 0.04 | 0.17 |
| *unclassified* | 1.39 | 1.96 | 1.17 | 2.20 | 1.80 | 1.78 |
| **Planctomycetes** | | | | | | |
| *MVS-107* | 0.35 | 0.05 | 0.40 | 0.95 | 0.00 | 0.00 |
| *d113* | 0.00 | 0.00 | 0.05 | 0.00 | 0.58 | 0.00 |
| *CL500-15* | 0.08 | 2.54 | 0.53 | 0.84 | 0.09 | 0.09 |
| *agg27* | 0.34 | 2.54 | 0.45 | 0.98 | 1.39 | 1.81 |
| *AKAU3564* | 0.00 | 0.11 | 0.00 | 0.00 | 0.00 | 0.00 |
| *CCM11a* | 0.01 | 0.22 | 0.02 | 0.03 | 0.27 | 0.60 |
| *Cpla-3* | 0.96 | 2.00 | 1.82 | 2.82 | 2.90 | 0.95 |
| *MSBL9* | 0.00 | 0.00 | 0.00 | 0.00 | 0.03 | 0.00 |
| *Phycisphaerales* | 1.83 | 1.14 | 1.71 | 3.66 | 2.41 | 2.42 |
| *Pla1* | 0.07 | 0.00 | 0.09 | 0.06 | 0.00 | 0.00 |
| *S-70* | 0.14 | 0.32 | 0.12 | 0.56 | 0.08 | 0.00 |
| *WD2101* | 15.97 | 10.55 | 16.85 | 13.49 | 7.77 | 13.30 |
| *B97* | 0.11 | 0.27 | 0.38 | 0.92 | 0.70 | 2.16 |
| *Gemmatales* | 57.64 | 43.40 | 53.55 | 46.70 | 53.35 | 39.81 |
| *Pirellulales* | 10.46 | 20.73 | 13.23 | 18.07 | 21.42 | 28.15 |
| *Plantomycetales* | 9.98 | 9.42 | 8.28 | 7.82 | 5.60 | 7.25 |
| *DH61* | 0.29 | 0.27 | 0.66 | 0.25 | 0.31 | 0.00 |
| *p04_C01* | 0.01 | 0.43 | 0.60 | 1.42 | 0.48 | 0.17 |
| unclassified | 1.78 | 6.01 | 1.27 | 1.42 | 2.64 | 3.28 |
| **β-Proteobacteria** | | | | | | |
| *A21b* | 4.08 | 4.14 | 3.15 | 4.68 | 0.44 | 7.84 |
| *Burkholderiales* | 49.23 | 78.00 | 39.20 | 39.93 | 23.66 | 35.59 |
| *Ellin6067* | 0.36 | 0.00 | 3.72 | 2.72 | 5.70 | 6.91 |
| *Gallionellales* | 3.42 | 0.00 | 0.06 | 0.00 | 37.08 | 18.99 |
| *IS-44* | 7.83 | 2.54 | 2.77 | 2.89 | 0.07 | 0.00 |
| *MND1* | 12.80 | 0.46 | 16.23 | 5.45 | 2.54 | 0.00 |
| *Methylophilales* | 1.01 | 9.03 | 2.92 | 2.17 | 1.67 | 2.26 |
| *Neisseriales* | 0.60 | 0.08 | 0.52 | 0.60 | 1.06 | 0.80 |
| *Nitrosomonadales* | 0.10 | 0.08 | 0.06 | 0.00 | 0.85 | 0.13 |
| *Procabacteriales* | 0.13 | 0.23 | 0.20 | 0.13 | 1.36 | 1.33 |
| *Rhodocyclales* | 0.11 | 0.15 | 0.10 | 0.00 | 1.73 | 1.06 |
| *SC-I-84* | 11.21 | 3.57 | 12.44 | 13.62 | 11.75 | 7.84 |
| unclassified | 9.13 | 1.75 | 18.61 | 27.80 | 12.09 | 17.26 |

**Supplementary Table S4**. Percent of initial biochar lost from biochar litterbags over four years at each of the three study sites. Values represent mean ± SEM for four replicates.

|  | **7 months** | **12 months** | **18 months** | **2 years** | **3 years** | **4 years** |
| --- | --- | --- | --- | --- | --- | --- |
| HF upslope | 4.71 ± 0.37 | 4.76 ± 0.22 | 4.69 ± 0.73 | 4.30 ± 0.27 | 7.56 ± 0.55 | 6.10 ± 0.73 |
| HF downslope | 8.62 ± 0.78 | 9.03 ± 0.97 | 8.61 ± 1.15 | 6.67 ± 2.06 | 9.54 ± 1.43 | 6.28 ± 1.41 |
| UTM | 10.7 ± 2.62 | 9.95 ± 6.91 | 12.8 ± 7.19 | 13.4 ± 7.49 | 9.75 ± 5.63 | 20.4 ± 16.5 |

**Supplementary Table S5**. Primer sequences used in this study.

| 515f | GTGCCAGCMGCCGCGGTAA |  |
| --- | --- | --- |
| 806r | GGACTACHVGGGTWTCTAAT |  |
| 7f | AACCTGGTTGATCCTGCCAGT |  |
| 570r | GCTATTGGAGCTGGAATTAC |  |
